# Can the asymmetry of Okun's law be interpreted from a social perspective? Evidence from the World Bank

**DOI:** 10.3389/fpubh.2022.1052812

**Published:** 2022-12-01

**Authors:** Lin Yi, Song Kaifeng, Tu Jingbin, Lai Liangrong

**Affiliations:** School of Business, Guangxi University, Nanning, China

**Keywords:** Okun's law, asymmetry, unemployment rate, alcohol consumption, economic growth

## Abstract

Okun's law is generally interpreted in economic terms. This paper is interpreted from a social perspective through regression and mediating effect models. Okun's law is demonstrated based on data from the World Development Indicators (WDI) dataset. There is indeed a relationship between unemployment and economic growth, with unemployment negatively affecting economic growth after controlling for the two-way causality problem. This result remains robust after replacing the core explanatory variables. This paper interprets the asymmetry of Okun's law from a social perspective, using alcohol consumption as a mediating variable to measure the harm caused by unemployment to the unemployed. Analysis of the data reveals that alcohol consumption mediates between unemployment and economic growth. Unemployment increases alcohol consumption, which causes incurable harm to the unemployed. It reduces the willingness and probability of the unemployed to re-employ and further reduces the potential for economic growth. This is why the economic expansion did not lead to employment growth, revealing the asymmetry of Okun's law. The gender-specific heterogeneity analysis found that the impact of unemployment on economic growth did not vary much by gender.

## Introduction

Full employment and economic growth are essential objectives of macroeconomic policy regulation and are one of the main concerns of modern macroeconomic research. In the complex relationship between economic growth and employment, the combination of “high growth and low unemployment” has always been the governing goal of governments worldwide. The “negative correlation between economic growth and unemployment” reflected in Okun's law provides the theoretical basis and practical possibility for this governing goal. In 1962, the American economist Okun observed a stable relationship between economic growth and unemployment rates, a relationship known as “Okun's Law.” Empirical data from several countries have confirmed this relationship (1, 2). As the economy evolves, the growth rate and the unemployment rate are not a constant ratio, i.e., the correspondence between the economic growth rate and the unemployment rate in some years is not in line with Okun's law (3, 4). The reasons are attributed to changes in institutional conditions that affect rigidities or lead to distortions related to efficiency wages, unionization and employee protection, wage contracts, unemployment insurance, labor force participation, or productivity (4, 5). In recent years, the study of Okun's law has formed two branches. One is the extension from linear to non-linear laws (6, 7), and the other is the extension of the univariate variables used in the law to panel data situations (8). All of these studies have significantly broadened the content of Okun's law and provided guidance to global economic development. However, unemployment and economic growth involve all aspects of society, and can Okun's law be interpreted only from an economic perspective?

**Figure 1:**
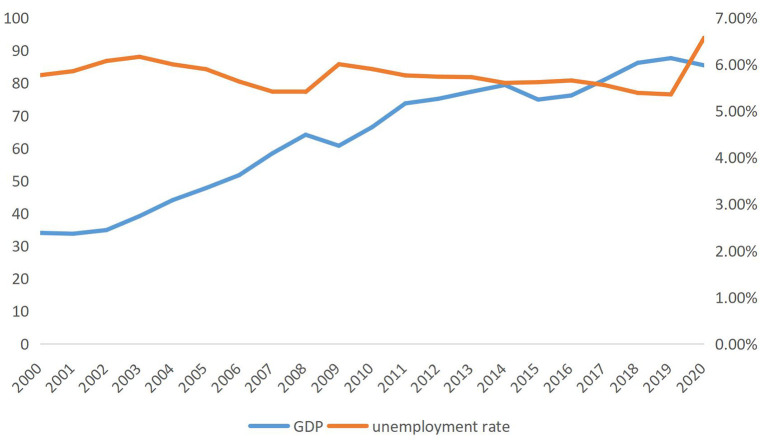
World gross product (at current prices) and unemployment rate (modeled ILO estimates: % of the total labor force). Data source: CEIC database.

Since the 21^st^ century, the global economy has shown a higher quality of development and provided many quality labor jobs due to the deepening of international free trade and the requirements of the Sustainable Development Goals. However, global job market is also facing many pressures and challenges. With the global economic downturn, frictional and structural unemployment has become a risk that most people need to face and a plight that some are experiencing (see Figure 1). In 2000, the Internet bubble bursting led to a steady climb in global unemployment. By 2003, the global unemployment rate was as high as 6.16%. Then, as the economy recovered, the unemployment rate declined from 2004 to 2008. The impact of the US subprime mortgage crisis saw global unemployment rise again, with the global unemployment rate reaching 6.01% in 2009 and global economic growth weakening. Subsequently, although unemployment rates have fallen in some developed countries, analysis shows that the global unemployment crisis is still ongoing. In particular, the global job market has been hit hard by the impact of COVID-19. The International Labor Organization (ILO) expects the global unemployment rate to reach 5.7% in 2022 and the number of unemployed people worldwide to reach 205 million, well above 187 million in 2019^1^

Economic growth is a universal pursuit for all countries. After the world economy fell into recession in 2000, it started to recover slowly from the bottom in 2002, and the global economy was on a steady growth track. In 2008, the subprime mortgage crisis occurred in the United States. It quickly triggered a domino effect that hit countries' economies worldwide, especially developing countries. From 2008 to 2021, the average annual growth rate of the world economy dropped to 3.4%, and it was slow to emerge from its weak state (9). Since the spread of COVID-19, some global industrial chains have encountered bottlenecks due to embargo management. According to International Monetary Fund (IMF) data, the world economy shrank by 3.3% in 2020, and economic growth entered a period of contraction^2^ The world economy faces severe challenges, from high inflation, fragile supply chains, and energy and food crises.

Against the above background, this paper is dedicated to a more in-depth interpretation of Okun's law. According to Okun's law, there is a natural link between economic growth and unemployment. A vibrant economy may lead to more jobs, reducing the incidence of unemployment. Full employment means more labor creation, which may lead to faster economic growth. At the same time, there is an asymmetric effect of economic expansion and economic contraction on the unemployment rate (10), for which a great deal of previous research has been done. Past studies have focused on the impact of economic growth on unemployment. However, how does the unemployment rate act on economic growth? Especially in the era of rapid development of the Internet economy, more and more diversified forms of employment have emerged. Most people are no longer bound to traditional forms of employment, and there are more and more flexibly employed people. What form does unemployment take on economic growth, and what impact does it have on society Therefore, it is necessary to assess the impact of the unemployment rate on economic growth and the relationship between alcohol consumption and the unemployment rate and economic growth. Considering the relationship between economic growth and the environment, as discussed by Adebayo et al. (11) and Alola and Adebayo (12), the confounding effect of variables such as carbon emissions on the model was also considered for the study.

The innovation of this paper is reflected in the following two aspects. First, previous studies have mainly examined the impact of economic growth on the unemployment rate. This study changes the perspective of traditional research concerns and focuses on the impact of the unemployment rate on economic growth. Secondly, existing studies have generally interpreted asymmetry of Okun's law from the economic perspective, while the interpretation of Okun's law at the social level has been neglected. Therefore, this paper selects alcohol consumption as a mediating variable to explore its relationship between unemployment and economic growth. This paper broadens the perspective of Okun's law. The paper is structured as follows: Part 1 is an introduction; Part 2 conducts a literature review and presents the research hypotheses; Part 3 presents the data sources and model setting; Part 4 presents the results of the analysis between unemployment and economic growth; Part 5 further analyses the relationship between alcohol consumption in unemployment and economic growth; Part 6 concludes the study and presents policy recommendations, research shortcomings and outlook.

## Literature review and research hypothesis

### Unemployment rate and economic growth

Full employment and economic growth are the focus of modern macroeconomic research, and many scholars have studied them from various perspectives, such as ecology, energy, culture, and security (11, 13–18). As far as the relationship between unemployment and economic growth is concerned, the famous American economist Okun pointed out that there is a fixed quantitative relationship between economic growth and unemployment. Specifically, for every 2% decline relative to potential GDP, the unemployment rate rises by 1% (19). However, the simple premise of Okun's law as a theoretical expectation makes it only partially consistent with the reality of economic performance. Subsequent researchers have used data to test Okun's Law empirically. Moosa ran a linear regression using unemployment and economic growth data for seven developed G7 countries. The results showed that Okun's Law was significant, but Okun's Coefficient differed across countries (20). Kaufman also compared the output elasticity of unemployment across six countries, and the study came to similar conclusions (21). Using short-term unemployment data for 20 developed economies since 1980, Ball et al. show that Okun's law holds for most countries and is pretty stable over time but that Okun's coefficient varies considerably across countries (22, 23). There is a quantitative relationship between unemployment and economic growth based on the differences in the level of economic development, labor market rigidity, working hours, and productivity across countries. However, this relationship is flexible across countries. Okun's coefficient also varies between countries that do not use the level of economic development. Okun's coefficient for developing countries is about half that of developed countries and is more applicable in developing countries (22).

Because of the inconsistency of Okun's coefficient, scholars have begun to pay attention to the differences and effectiveness of Okun's coefficient in different countries, industries, and times. In Nigeria, Okun's law is significant in the short run but not in the long run (24). Researchers have also found a range of deviations and failures of Okun's Law in China (25, 26). On the one hand, this may be due to the failure of statistical data to accurately reflect the actual unemployment rate in China. On the other hand, it may be due to a large number of laid-off workers due to the reform of state-owned enterprises and the large number of migrant workers moving into the cities. Peijiang argues that simple Okun's law does not exist in China due to the inadequate market system and dualistic structure. However, Okun's law under a dualistic economic structure derived from the C-D production function still holds in China, where a 1% increase in the registered urban unemployment rate in China will lead to a 1.98% decrease in real GDP growth (27). Valadkhani's empirical results validate the validity of Okun's law in Australia using quarterly data from the third quarter of 1980 to the first quarter of 2014 (28). Karadzic et al. used an error-corrected model (ECM) to quantify the relationship between unemployment and economic growth rates. In Montenegro, for every 1% increase in economic growth, the unemployment rate will decrease by 0.27% in the short term and 1.46% in the long term. Okun's law is still in force in Montenegro (29). In India, the relationship between unemployment and output is consistent with Okun's law. The empirical results show that to reduce the unemployment rate by 1%, India would need a 25% nominal GDP growth rate (30). In Saudi Arabia, GDP has a negative and significant impact on the unemployment rate, with a 1% increase in GDP associated with a 0.29% decrease in the unemployment rate and the existence of Okun's law in the Saudi economy (31) Guisinger et al. study Okun's coefficient variation across US states and find that Okun's coefficient also varies across states, with education level and non-manufacturing employment being essential determinants of the variation in Okun's coefficient (32). Hartwig finds that Okun's law still holds in most sectors in Switzerland, with a one percentage point increase in the economy leading to a 0.3–0.4 percentage point increase in employment growth in the same quarter. However, in sectors such as energy and water supply, hotels and restaurants, banking, and health care, the law does not apply (2). Apap et al. find different sensitivities of the unemployment rate to services and manufacturing output in Malta and that Okun's coefficient is higher for services than for manufacturing (33). In the case of external shocks, Okun's coefficient also varies across time in a country. Using Okun's coefficient for Macedonia from 2004 to 2016 as a sample, Andonova et al. found that the global financial crisis and the employment-intensive growth resulting from the European debt crisis had reduced Okun's coefficient. However, in Poland, Okun's coefficient increased over time due to the widespread use of temporary contracts rather than permanent contracts in Polish companies, which made working relationships less secure and thus led to an increase in the Okun's coefficient (34). The effect of using temporary contracts on Okun's coefficient has also been demonstrated in Mexico (35). In the US, Okun's law also has time fluctuations. The main factors contributing to this fluctuation are working hours and productivity (36). In summary, there is a negative relationship between unemployment and economic growth. Therefore, hypothesis 1 is formulated.

**Hypothesis 1:** The unemployment rate negatively affects economic growth.

### The asymmetry of unemployment and economic growth

Okun's coefficient is also asymmetric and non-linear. Using data from the US Bureau of Labor Statistics as a sample, Lim finds that the long-run Okun's coefficient is 0.61 in a contracting economy and 0.24 in an expanding economy (37). Widarjono uses a non-linear autoregressive distributed lag (NARDL) and asymmetric pooled mean group (PMG) model to test for cointegration in the case of Malaysia, the Philippines, and Singapore. The results show an asymmetry in the Austrian law and that economic downturns impact unemployment more than economic booms (38). Mihajlovic uses the example of South East Europe, where Okun's coefficient has an asymmetric effect in five out of eight countries, suggesting that the unemployment effect is more pronounced in economic downturns than in upturns (39). This is supported by the data for Turkey and Mexico (40, 41).

There is an asymmetry in the interrelationship between unemployment and economic growth. Specifically, unemployment falls less due to economic growth than it rises due to economic contraction. In other words, the damage to unemployment during a contraction is greater than the benefit to unemployment during a growth period. The possible reason is a mismatch between the sectors and regions where employment and unemployment are located (42). When the economy shrinks, enterprises do not need to consider regional issues in layoffs. However, when the economy expands, people with employment needs may be outside the area where the enterprise with jobs is located, which leads to the demand for employment not translating into actual employment. Another possible explanation lies in the microeconomic asymmetry of adjustment costs (43). In a contracting economy, firms only have to bear the cost of laying off staff. In contrast, they have to bear the additional costs of expanding their plants, acquiring equipment, and increasing staff wages in an expanding economy. This leads to a willingness to cut jobs when the economy is contracting. When the economy is expanding, high levels of fixed asset investment may discourage willingness to expand. Companies also tend to maintain their current scale rather than expand production based on fears of recession. Moreover, when the economy is expanding, firms need time to adjust their staffing levels in the face of demand shortages. They tend to adjust hours and capacity utilization per worker first rather than adding workers (44).

All of the above explanations are economical and focus on the impact of economic development on the unemployment rate. Nevertheless, unemployment is not just an economic phenomenon, it is also a social phenomenon. Unemployment has a social cost. Unemployment increases crime rates, affects social order (16, 45), and creates mental health problems and suicidal tendencies for the unemployed (18, 46). Moreover, the impact of unemployment is wider than the period of unemployment. The damage caused by unemployment will continue to accompany people, affecting their income after secondary employment (47). Therefore, this paper explains the asymmetry between unemployment and economic growth from a social perspective. Unemployment takes a toll on the unemployed, both psychologically and physically. It reduces the probability of re-employment and the willingness of the unemployed to re-enter the workforce. This makes economic growth a lousy driver of employment and further reduces economic growth rate. The harm caused by unemployment can be reflected in crime, psychological problems, and physical health problems. However, crime as a small probability event is difficult to observe, and psychological and physical health problems are difficult to find relevant data to support. Considering both theoretical and practical possibilities, this paper uses alcohol consumption to measure the harm caused by unemployment. Alcohol can numb one's nerves, making people suffering hardship inclined to use it to relieve pain. Studies have shown that around 27% of suicide victims had a blood alcohol concentration above zero at the time of death (46). Many people try to use alcohol to relieve stress. There is a strong association between unemployment and alcohol consumption. Unemployment has a significant contribution to drinking behavior (48), and this contribution is outstanding in the case of long-term unemployment (49). Unemployed people are also more likely to suffer from alcohol than workers (50). Therefore, alcohol consumption plays an intermediary role in the relationship between unemployment and economic growth, which is also one of the sources of asymmetry between unemployment and economic growth. Hypothesis 2 is formulated.

Hypothesis 2: Alcohol consumption mediates the relationship between the unemployment rate and economic growth.

## Data sources and model setting

### Data sources

The data is derived from publicly available data published by the World Bank, and the indicators covered in this paper are taken from the World Development Indicators (WDI) dataset. The data spans the period 2000–2020 and includes 266 countries and territories, not only at the national and regional levels but also at the regional level, such as the EU, Africa, North America, and ASEAN.

The economic growth rate is the explanatory variable, measured by the GDP growth rate, and the unemployment rate is the explanatory variable, measured by the number of unemployed as a percentage of the total labor force (31). The control variables include the cost of time to start a business, the size of the economy, investment in innovation capital, investment in innovation personnel, job market inclusiveness, carbon emissions, electricity consumption, internet coverage, and health service capacity (See Table 1). If an economy is an organism, then firms are the cells that make up the organism, and the time it takes to start a business is the time it takes for the cells to regenerate, which reflects the dynamism of economic growth (51). The size of the economy is measured by the total number of companies listed in the country, reflecting the size of the current economy (52). Innovation is one of the driving forces of economic growth and is closely related to economic growth, so R&D expenditure and R&D research are used to measure innovation investment (53). Job market inclusiveness reflects the friendliness of the job market, which is undeniably gender-discriminatory in most countries. Women make up half of the population, and the female employment rate indicates the extent to which an economy is inclusive and in need of labor. The female employment rate can be a good indicator of the extent to which an economy is inclusive and needs labor (54). Economic growth is not only a labor input but also a resource input, especially in a modern economy where energy consumption is essential. Carbon emissions represent fossil energy consumption, while electricity consumption represents non-fossil energy consumption (55–57). Internet coverage and healthcare capacity reflect the extent to which infrastructure is available. Internet coverage can be measured by mobile phone subscriptions and fixed broadband Internet subscriptions. However, given the widespread popularity of mobile phones and the difficulty of having some heterogeneity between countries, fixed broadband Internet subscriptions were chosen to measure Internet coverage (58). Infants are physically weak and highly susceptible to disease. The treatment of infants tests the capacity of the health service, and therefore the infant mortality rate is used to measure the capacity of the health service (59).

**Table 1:** Description of variables.

**Variable classification**	**Variables**	**Explanation of variables**
Explained variables	Economic growth	GDP growth rate (annual %)
Explanatory variables	Unemployment rate	Total unemployment rate (% of total labor force)
Control variables	Time cost of starting a business	Time required to start a business (days)
	Size of the economy	Total number of domestic listed companies (logarithmic)
	Innovation funding	R&D expenditure (% of GDP)
	Innovative staff input	R&D researchers (find logarithm)
	Job market inclusiveness	Female employment rate (15+ years, %)
	Carbon emissions	Carbon dioxide emissions (metric tons per capita)
	Electricity consumption	Electricity consumption (kWh per capita, to the logarithm)
	Internet coverage	Fixed broadband internet users (find logarithm)
	Medical service capacity	Number of infant deaths (find logarithm)
Intermediate variables	Alcohol consumption	Total alcohol consumption per capita (projected pure alcohol over 15 years, liters)
Tool variables	Credit coverage	Public credit system coverage (% of adults)
	Level of civic education	Completed at least upper secondary education, % of population aged 25+

### Model setting

A Two-way fixed effects model is used to test the impact of unemployment on economic growth. In the panel data, we introduce the concept of fixed effects. Individual fixed effects refer to considering the trend characteristics that individuals exhibit in the absence of the intervention, then controlling for such trend characteristics in the treatment and control groups, and finally comparing the difference in levels between the two. However, there may also be the problem of omitted variables that do not vary with individuals and over time, when a time-fixed effects model needs to be considered. When both personal and temporal fixed effects are considered, this is known as a “two-way fixed effect,” i.e., controlling for individual characteristics that do not change over time and individuals that do not change over time.

A random effects model must be considered before choosing a Two-way fixed effects model. A fixed effects model considers individual characteristic variables to be explanatory variables, while a random effects model takes individual characteristic variables into account in the random error term. This is why the explanatory variables in the fixed effects model can be correlated with the individual characteristics variables but not in the random effects model. We need to make a trade-off between the two. The criterion for this trade-off is the result of the Hausman test. If The Hausman test results in a significant difference in the coefficient estimates, the fixed-effects model is chosen.

**Figure 2:**
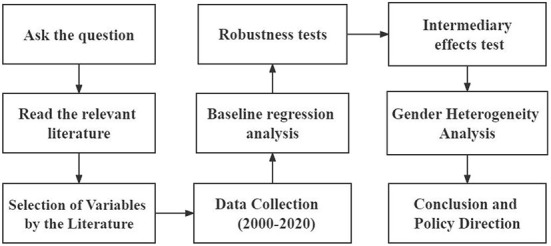
Flow of the analysis.

In order to interpret the asymmetry of Okun's law, the paper uses alcohol consumption as a mediating variable. Unemployment brings harm to workers, thus reducing their probability of re-employment and their willingness to re-employ, making economic growth not well-driven by employment, specifically manifesting itself as the asymmetry of Okun's law. According to scholars Wen et al., Consider the effect of the independent variable X on the dependent variable Y. If X affects Y by influencing the variable M, then M is said to be the mediating variable (60). The mediating role of alcohol consumption is also tested by drawing on the Sobel mediating factor effect model. The mediating role of alcohol consumption was tested using the Sobel mediating factor effects model. In addition, Figure 2 presents an analysis diagram.

## Results of the empirical analysis

### Baseline regression results

The original hypothesis of “no individual random effects” was firmly rejected. Therefore, the random effects model should be chosen. The panel fixed effects regressions were tested with an F-test, and the fixed effects model was also found to be better than the mixed effects model. Therefore, we chose fixed-effect and random-effects models. Since the Hausman test has a negative test statistic, a negative value can be considered a rejection of the original hypothesis of “using a random effects model,” probably because the original hypothesis of random effects was not met (61). The negative value can be considered a rejection of the original hypothesis of “using a random effects model.” Therefore, the fixed effects model was used for estimation. Finally, a time effects test was conducted to consider the possible existence of time effects. The results concluded that the model should include time effects, so a two-way fixed effects model was used to estimate the coefficients. However, estimates are also reported for mixed, random, and fixed effects to indicate the robustness of the regression results. See Table 2 for details.

**Table 2:** Impact of unemployment on economic growth.

**Variables**	**(1)**	**(2)**	**(3)**	**(4)**
	**Mixed effects**	**Random effects**	**Fixed effects**	**Two-way fixed effect**
Unemployment rate	−0.22^***^	−0.236^***^	−0.25^***^	−0.24^***^
	(−6.56)	(−5.33)	(−3.27)	(−4.32)
Time cost of starting a business	−0.001	−0.002	−0.004	0.005
	(−0.24)	(−0.02)	(−0.31)	(0.56)
Size of the economy	0.16	0.072	−0.51	−0.29
	(1.25)	(0.35)	(−1.02)	(−0.81)
Innovation funding	−0.18	−0.60	−3.60^***^	−1.37^**^
	(−0.70)	(−1.41)	(−3.82)	(−1.98)
Innovative staff input	0.067	0.26	2.21^**^	1.19^*^
	(0.27)	(0.69)	(2.45)	(1.83)
Job market inclusiveness	−0.016	−0.024	−0.11	0.025
	(−0.89)	(−0.88)	(−1.20)	(0.36)
Carbon emissions	0.042	0.021	−0.047	−0.44^**^
	(0.85)	(0.27)	(−0.18)	(−1.99)
Electricity consumption	0.46	0.89	7.10^**^	3.03
	(0.99)	(1.16)	(2.30)	(1.35)
Internet coverage	−1.11^***^	−1.12^***^	−1.40^***^	−1.24^***^
	(−9.68)	(−8.25)	(−6.12)	(−5.72)
Medical service capacity	0.75^***^	0.79^***^	−0.13	−0.78
	(6.32)	(4.14)	(−0.08)	(−0.54)
_cons	10.62^***^	6.85	−36.19	−0.15
	(3.47)	(1.37)	(−1.13)	(−0.23)
N	599	599	599	599
Prob > chi 2 or F	0.00	0.00	0.00	0.00

T-values in brackets,

*, **, *** indicate significant at the 10, 5 and 1% statistical levels, respectively.

The results in Table 1 show that the coefficients of the two-way fixed effects model are negative and significant at the 1% level. The estimates of mixed effects, random effects, and fixed effects are also significantly negative. Unemployment significantly and negatively affects economic growth. Hypothesis 1 is tested. This finding validates the theoretical assertion that unemployment negatively affects economic growth, as given in Okun's law. Moosa and Kaufman et al. reported similar results (20, 21). For specific reasons, some countries and regions, such as China, may also have deviations or errors in Okun's law (26, 27). As for the control variables, the input of innovators significantly and positively affects economic growth. Innovation is one of the driving forces of economic development, and the input of innovators stimulates innovation and enhances the country's science and technology level, thus promoting economic growth. However, financial investment in innovation's impact on economic growth is insignificant. This may be because innovation is a long-term process focusing on innovators. The effect of financial investment on funding and motivating innovators needs a long-term process to become apparent. Working in the short term is challenging, and even the diversion of funds negatively affects economic growth in the short term. Carbon emissions significantly and negatively affect economic growth, while electricity consumption positively affects economic growth but not significantly. This suggests that environmental friendliness is one of the paths to maintaining high economic growth. Internet coverage significantly negatively impacts economic growth, which is outside research expectations. The possible reason is that countries with high internet coverage are generally developed economies, which generally do not have high economic growth rates.

### Robustness tests

#### Two-way causal issues

Two-way causality is a prominent issue in the relationship between unemployment and economic growth. A high unemployment rate can inhibit economic growth. A macroeconomic slump during an economic contraction can also reduce jobs and thus increase unemployment. The instrumental variables approach can be an excellent solution to this problem. The instrumental variables approach must be correlated with the explanatory variables and uncorrelated with the nuisance terms to satisfy the homogeneity requirement. Therefore, credit coverage and citizens' educational attainment were selected as instrumental variables from the requirements of relevance and homogeneity. First, unemployment causes citizens to take on debt and thus join the public credit system, and the level of education is also closely related to the unemployment rate. In contrast, economic growth in the current period hardly affects credit coverage and citizen education in the short term, satisfying the exogenous requirement.

Table 3 reports the results of the two-stage least squares (2SLS) estimation using the instrumental variables method. Column 1 presents the results of the first stage regression, where both the instrumental variables credit coverage and civic education are significantly related to the unemployment rate, satisfying the correlation hypothesis. The unidentifiability test shows a high degree of rejection of the original hypothesis and that the model is not unidentifiable. The minimum eigenvalue of the weak instrumental variable is 14.08, which is greater than the corresponding critical value of 11.59, rejecting the original hypothesis of the weak instrumental variable and justifying the conclusion that there is no weak instrumental variable problem. The Sargan statistic of the over-identification test is 0.093, accepting the original hypothesis that all instrumental variables are exogenous. In summary, the instrumental variables chosen can be considered reasonable. The regression results at stage 2 show that the coefficient of the effect of unemployment on economic growth remains negative and is statistically significant at the 1% level. After eliminating the two-way causality problem that exists, the unemployment rate remains a significant inhibitor of economic growth.

**Table 3:** The impact of unemployment on economic growth: An instrumental variables approach.

**Variables**	**(1)**	**(2)**
	**Unemployment rate**	**Economic growth**
Unemployment rate		−0.43^***^
		(−2.96)
Instrumental variable 1	0.056^***^	
(credit coverage)	(4.32)	
Instrumental variable 2	0.095^***^	
(educational attainment of citizens)	(4.85)	
Control variables	yes	yes
_cons	7.28	14.25^***^
	(1.04)	(2.63)
Chi-sq(2) P-val		26.40
C-Donald Wald F		14.08
Chi-sq(1) P-val		0.093
N	243	243
Prob > F	0.00	0.00

T-values in brackets,

*, **, *** indicate significant at the 10, 5 and 1% statistical levels, respectively.

#### Replacement of core explanatory variables

Due to the greater adaptability of young people and the diversity of employment, youth unemployment may have a minor impact on economic growth than expected. The youth unemployment rate (total number of unemployed youth as a percentage of the total labor force aged 15–24) was estimated by replacing the total unemployment rate, and the results are shown in Table 4. It can be seen that after replacing the core explanatory variables, the regression coefficient drops from −0.24 to −0.11. The advantages that young people possess do make their unemployment less influential on economic growth but remain statistically significant, reflecting the unemployment rate's negative impact on the robustness of the negative impact of unemployment on economic growth. The results are similar to those of Blázquez Fernández et al. (62) and Zanin (63).

**Table 4:** Robustness tests: Replacing core explanatory variables.

**Variables**	**(1)**		**(2)**
	**Random effects**	**Fixed effects**	**Two-way fixed effect**
Youth unemployment rate	−0.12^***^	−0.11^***^	−0.11^***^
	(−5.66)	(−3.03)	(−4.06)
Control variables	yes	yes	yes
_cons	8.92^*^	−26.28	21.13
	(1.76)	(−0.76)	(0.86)
N	588	588	588
Prob > F	0.00	0.00	0.00

T-values in brackets,

*, **, *** indicate significant at the 10, 5 and 1% statistical levels, respectively.

## Further analysis

### Asymmetry in unemployment and economic growth

The asymmetry of Okun's law suggests that the increase in unemployment brought about by an economic expansion is lower than the decrease in unemployment caused by a contraction. Possible reasons for this have been explored from an economic perspective. This paper explains the asymmetry of Okun's law from a social perspective. Unemployment affects the current income of the unemployed and causes permanent damage to them, reducing their willingness to re-enter the workforce. Even more, the long-term disconnection from the job market reduces the probability that the unemployed will re-enter the workforce. As a result, when the economy improves, the potential demand for labor does not translate well into actual employment and further pulls down economic growth.

Table 5 reports the test results for the mediating role of alcohol consumption in the effect of unemployment on economic growth. Path A results show that unemployment significantly negatively affects economic growth. Path B results show that unemployment is significantly positively related to alcohol consumption. After including the mediating factor, the results of path C show that unemployment has a non-significant effect on economic growth. Alcohol consumption significantly and negatively affects economic growth and has a significant Sobel Z value, thus accounting for a mediating effect of 33%. This suggests that alcohol consumption has a fully mediating role in the effect of unemployment on economic growth, and hypothesis 2 is tested. This result changes the traditional research perspective and validates Okun's law from a social perspective (28, 29). When the unemployed lose their jobs, their current income decreases, yet they have to spend money on all kinds of goods, such as food, oil, and salt, to live. The saying goes, “a poor couple is a poor couple.” Unemployment may lead to further family conflicts. Unemployed people suffering from social and family conflicts use alcohol to numb themselves, and alcohol abuse takes a physical and psychological toll on the unemployed. Even if jobs exist, some unemployed people may find it difficult to break free from their plight and return to work. The potential demand for labor needs to be met, inhibiting economic growth to some extent.

**Table 5:** Results of the Sobel mediating factor effect test.

**Variables**	**Path A**	**Path B**	**Path C**
	**Economic growth**	**Alcohol consumption**	**Economic growth**
Unemployment rate	−0.098^*^	0.17^**^	−0.066
	(−1.65)	(2.42)	(−1.10)
Alcohol consumption			−0.19^**^
			(−2.56)
Control variables	yes	yes	yes
_cons	12.03^***^	−13.73^**^	9.41^**^
	(2.65)	(−2.55)	(2.06)
N	132	132	132
adj R^2^	0.21	0.51	0.24
Prob > F	0.00	0.00	0.00
Sobel Z values absolute	−1.75^*^		
Intermediary effect as a percentage	0.33		

T-values in brackets,

*, **, *** indicate significant at the 10, 5 and 1% statistical levels, respectively.

### Impact of gender-specific unemployment rates on economic growth

There may be differences in the impact of male and female unemployment rates on economic growth due to differences in male and female attitudes to unemployment and social roles. Therefore, the adverse effects of male and female unemployment on economic growth are analyzed separately. Firstly, unemployed women may be more likely to return to their families and take on small part-time jobs while dealing with household chores, which can be difficult for men to accept. Thus, the negative effect of male unemployment on economic growth may be more significant in relative terms. Secondly, the difference in social roles may make unemployment more damaging for men. Men are generally expected to be the breadwinners of their families, and unemployment makes it difficult for men to meet the expectations of their families and society. Unemployment exposes men to more significant hardship, leading to demotivation, malaise, and a further reduction in their contribution to economic growth. On the other hand, the expectations of men in terms of social roles may lead men to seek job opportunities and informal employment after unemployment. The negative effect of male unemployment on economic growth may be less significant.

Table 6 reports the impact of unemployment rates on economic growth by gender. The results show that although the negative effect of unemployment rates on economic growth is more significant for men than women, the difference is not significant. The differences in male and female unemployment attitudes and social roles do not significantly affect the impact of unemployment rates on economic growth across genders. This is in line with the findings of Martin and Mariana's study (64), contrary to the results of Clark and Summers (65).

**Table 6:** Impact of gender-specific unemployment rates on economic growth.

**Variables**	**(1)**	**(2)**
Male unemployment rate	−0.18^***^	
	(−3.57)	
Female unemployment rate		−0.16^***^
		(−3.01)
Control variables	yes	yes
Time effect	yes	yes
_cons	−14	−9.35
	(−0.39)	(−0.42)
N	597	597
Prob > F	0.00	0.00

T-values in brackets,

*, **, *** indicate significant at the 10, 5 and 1% statistical levels, respectively.

## Conclusion, recommendations, and future directions

### Conclusion

Okun's law is demonstrated based on data from the World Development Indicators (WDI) dataset. There is indeed a relationship between unemployment and economic growth, with unemployment negatively affecting economic growth after controlling for the two-way causality problem. This result remains robust after replacing the core explanatory variables. This paper interprets the asymmetry of Okun's law from a social perspective, using alcohol consumption as a mediating variable to measure the harm caused by unemployment to the unemployed. Analysis of the data reveals that alcohol consumption has a mediating effect between unemployment and economic growth. Unemployment increases alcohol consumption, which causes incurable harm to the unemployed. It reduces the willingness and probability of the unemployed to re-employ and further reduces the potential for economic growth. This is why the economic expansion did not lead to employment growth, revealing the asymmetry of Okun's law. The gender-specific heterogeneity analysis found that the impact of unemployment on economic growth did not vary much by gender.

### Policy recommendations

In order to fully promote employment during economic expansion and reduce the harm to the unemployed, governments need to respond with more active measures. According to research findings, unemployment negatively affects economic growth, and governments must take active policy measures to reduce unemployment to ensure that the unemployment crisis does not worsen. For general frictional unemployment, governments should actively use internet technology and fully use social resources. For example, an online job information exchange platform should be established to complete the matching of enterprises and employees. For structural unemployment, which is long-term and inevitable, the government needs to strengthen skills training for the unemployed to adapt to more jobs; if the unemployed take unconventional forms of employment, the government needs to strengthen guidance to ensure a good employment environment. For cyclical unemployment, the government should, on the one hand, provide subsidies to encourage enterprises to expand production and increase the demand for labor; on the other hand, it can provide some infrastructure construction to provide jobs. Nowadays, countries worldwide are experiencing high unemployment on the one hand and high inflation on the other. Therefore, interest rates should be raised appropriately to suppress inflation while encouraging production and opening free trade to increase supply. Studies have found that youth unemployment has a much lower economic impact. Middle-aged people with older and younger children will significantly impact individual families and the national economy. The government can establish a system of categorizing and assisting the unemployed for different age groups. Targeted assistance can be provided by identifying the causes and characteristics of the unemployed.

We also make some policy recommendations for governments at the social level. Based on the relationship between alcohol consumption in terms of unemployment and economic growth, there may be better solutions than a purely expansionary fiscal policy. Firstly, as employment is a matter of livelihood and wellbeing, governments should introduce various measures to promote employment, such as free skills training for the unemployed. Free skills training for the unemployed acts as a reservoir of labor and allows the unemployed to acquire the necessary skills for employment. Employers are also more willing to hire people with skills. This is how restrictions on labor employment can be broken. Secondly, unemployment is a permanent injury to the unemployed. We may not be able to eliminate the harm caused by unemployment, but we can minimize the harm caused by unemployment as much as possible. More is needed to provide the necessary livelihood protection. Professionals should be encouraged to provide the necessary psychological counseling to the unemployed and to pay attention to the mental health of the unemployed.

### Limitation and future recommendation

The indicators (WDI) dataset is used to verify the asymmetry of the Austrian law from a societal perspective. However, the WDI dataset is sourced from each country's official system. It has many missing values due to differences in government systems, levels of governance, and statistical capacity in each country, as well as differences in the importance attached to this dataset by each country. This undoubtedly reduces the robustness and representativeness of the estimates. A particular country may have been dropped from the regression sample because there needed to be more data. Follow-up studies could be conducted at different levels using more complete data, for example, for individual countries. The paper uses alcohol consumption to measure unemployment's physical and psychological toll, a compromise made for data availability. Subsequent research could examine the permanent scars of unemployment directly in terms of physical and psychological damage, using data from other sources. While this study utilizes data at the macro level, subsequent studies could also examine the injury caused by unemployment and its impact on the probability of re-employment and willingness to re-employ at the micro level. There are differences in the impact of unemployment on economic growth by gender. Follow-up research could delve into whether there are significant differences in the effect of unemployment rates on economic growth by gender or the underlying mechanisms by which such differences are not significant.

## Data availability statement

The original contributions presented in the study are included in the article/supplementary material, further inquiries can be directed to the corresponding author.

## Author contributions

LY: methodology, investigation, supervision, and funding acquisition. SK: conceptualization, formal analysis, and resources. SK and TJ: writing—original draft. TJ: validation, visualization, submissions, and revisions. LL: English translation and guidance on the publication process of the thesis. All authors contributed to the manuscript revision, read, and approved the submitted version.

## Funding

This research was funded by the National Social Science Foundation of China (18BJL082), Guangxi Philosophy and Social Science Planning Research Subjects (22FGL039), and Key Research Base of Humanities and Social Sciences in Guangxi Universities in Guangxi Development Strategy Institute (2022GDSIQM18).

## Conflict of interest

The authors declare that the research was conducted in the absence of any commercial or financial relationships that could be construed as a potential conflict of interest.

## Publisher's note

All claims expressed in this article are solely those of the authors and do not necessarily represent those of their affiliated organizations, or those of the publisher, the editors and the reviewers. Any product that may be evaluated in this article, or claim that may be made by its manufacturer, is not guaranteed or endorsed by the publisher.

## Footnotes

1 https://www.ilo.org/global/lang–en/index.htm

2 https://www.imf.org/zh/home

## Abbreviations

Okun's law: Refers to a fairly stable relationship that exists between changes in GDP and changes in the employment rate
WDI: The World Development Indicators
COVID-19: Corona Virus Disease 2019
ILO: The International Labor Organization
IMF: International Monetary Fund
GDP: Gross Domestic Product
G7: Group of 7, including USA, UK, Germany, France, Italy, Canada, and Japan
C-D: Cobb-Douglas
ECM: Error-corrected model
NARDL: Nonlinear autoregressive distributed lag
PMG: Asymmetric pooled mean group model
ASEAN: Association of Southeast Asian Nations
R&D: Research and development
LM test: Lagrange multiplier test
F-test: Joint hypothesis testing
2SLS: Two-stage least squares.

## Appendix

In order to verify the effect of the unemployment rate on economic growth, this paper refers to Yang and Hou's study. It uses a double fixed effects model for regression analysis (66). The model is as follows.

(1)
$$\begin{array}{l} gdp_{i}=\beta_{0}+\beta_{1}\;unemployment_{i}\;+\;\gamma ‘\;control_{i}\;+\;\mu_{i} \end{array}$$

where *gdp*_*i*_ is the explanatory variable, representing economic growth. *unemployment*_*i*_ is the core explanatory variable, representing the unemployment rate. β_0_, β_1_, and β‘ denote the parameters to be estimated. β_0_ are the constant terms. β_1_ are the coefficients of the explanatory variables. β‘ is the vector of coefficients of the control variables, and *control*_*i*_ denotes the set of control variables for individual i, and ε_*i*_ is the error term.

In order to test the relationship between alcohol consumption on unemployment and economic growth, the mediating role of alcohol consumption was tested by drawing on the Wen Zhonglin Sobel mediating factor effect model (60). The specific path models Path a (Equation 2), Path b (Equation 3), and Path c (Equation 4) are set as follows.

(2)
$$\begin{array}{l} gdp_{i}=\alpha_{0}+\alpha_{1}unemployment_{i}+\gamma ‘control_{i}+\mu_{i} \end{array}$$

(3)
$$\begin{array}{l} {alcohol}_{i}=\beta_{0}+\beta_{1}unemployment_{i}+\gamma ‘control_{i}+\mu_{i} \end{array}$$

(4)
$$\begin{array}{l} gdp_{i}=\delta_{0}+\delta_{1}unemployment_{i}+\delta_{2}alcohol_{i}\\ \;+\gamma ‘control_{i}+\mu_{i} \end{array}$$

Alcohol consumption is fully mediated when the Sobel-mediated effects model regression coefficients α_1_, β_1_, and δ_2_ are significant. However, the regression coefficient δ_1_ is insignificant, and the Sobel Z value is statistically significant. Alcohol consumption is partially mediated when the regression coefficients α_1_, β_1_, and the regression coefficients δ_1_ and δ_2_ are all significant, δ_1_ is significantly smaller than α_1_, and the Sobel Z value is statistically significant.
